# Improving Long-Term Adherence to Monitoring/Treatment in Underserved Asian Americans with Chronic Hepatitis B (CHB) through a Multicomponent Culturally Tailored Intervention: A Randomized Controlled Trial

**DOI:** 10.3390/healthcare10101944

**Published:** 2022-10-05

**Authors:** Grace X. Ma, Lin Zhu, Wenyue Lu, Elizabeth Handorf, Yin Tan, Ming-Chin Yeh, Cicely Johnson, Guercie Guerrier, Minhhuyen T. Nguyen

**Affiliations:** 1 Center for Asian Health, Lewis Katz School of Medicine, Temple University, Philadelphia, PA 19140, USA; 2 Department of Urban Health and Population Science, Lewis Katz School of Medicine, Temple University, Philadelphia, PA 19140, USA; 3 Fox Chase Cancer Center, Temple University Health System, Philadelphia, PA 19111, USA; 4 Nutrition Program, Hunter College, City University of New York, New York, NY 10065, USA; 5 Hunter College Center for Cancer Health Disparities Research (CCHDR), Hunter College, City University of New York, New York, NY 10065, USA; 6 College of Science and Technology, Temple University, Philadelphia, PA 19122, USA

**Keywords:** chronic hepatitis b, Asian Americans, monitoring, treatment, intervention

## Abstract

Background: Although Asian Americans make up 6% of the U.S. population, they account for 58% of Americans with chronic hepatitis B (CHB). Yet, adherence to monitoring and antiviral treatment guidelines among Asian American CHB patients remains suboptimal. Methods: The purpose of this study was to evaluate the efficacy of a multicomponent intervention on adherence to CHB monitoring among Asian Americans with CHB. The intervention components included virtual patient education, patient navigation, and mobile health reminders delivered by bilingual community health educators. Chi-square test and *t*-test were used to compare demographic characteristics and two CHB measures: CHB clinical follow-up and CHB laboratory monitoring by the time of the 12-month follow-up assessment. A generalized linear mixed-effects model (GLMM) was fitted to assess the effectiveness of the intervention. Results: The study sample consisted of 358 Chinese and Vietnamese Americans living with CHB, including 181 in the intervention group and 177 in the control group. The intervention group had a significantly higher rate of CHB clinical follow-up (86.2%) and CHB laboratory monitoring (79.0%) than did the control group (54.2% and 45.2%, respectively). Results of the GLMM showed significant intervention effects on CHB clinical follow-up (odds ratio = 7.35, 95% confidence interval = 4.06–13.33) and CHB laboratory monitoring (odds ratio = 6.60, 95% confidence interval = 3.77–11.56) at the 12-month follow-up assessment. Conclusion: The multicomponent intervention was effective in improving adherence to CHB monitoring among Asian Americans. Additional implementation research is needed to better understand and apply effective interventions to other underserved populations.

## 1. Introduction

Although chronic hepatitis B (CHB) usually remains asymptomatic for decades, it ultimately can lead to life-threatening complications, including hepatocellular carcinoma (HCC) [1,2]. Individuals with CHB need regular follow-up and monitoring for disease progression, treatment adherence, and HCC risk. The current guidelines from the American Association for the Study of Liver Diseases (AASLD) recommend that individuals with CHB receive regular monitoring every six months with abdominal ultrasound and blood tests for liver function, HBV load, and alpha-fetoprotein (AFP) levels [3,4,5,6]. The purposes of these monitoring activities are to check for liver disease activity and to inform treatment decisions [3,4,5,6]. Cumulating clinical evidence has shown that long-term adherence to recommended CHB management guidelines can prevent complications associated with liver cancer treatments or transplants and reduce the risk of premature death [7,8,9,10].

Asian Americans are disproportionately affected by CHB. While they make up only 6% of the US population [11], they account for 58% of the 862,000 Americans living with CHB [12]. Despite the recommendations, adherence to HCC surveillance (12–28%) and treatment (16–32%) is suboptimal among Asian Americans, especially those with low socioeconomic status and limited English proficiency [13]. Multiple studies have suggested that adherence to CHB monitoring and follow-up care among Asian Americans is as low as 40–53% [14,15,16]. For example, in a study of two community clinics serving predominantly Asian Americans, researchers found that only 40% of CHB patients eligible for treatment returned for follow-up care within 12 months of receiving treatment recommendations [15]. Such suboptimal adherence to CHB monitoring and treatment resulted in delayed HCC diagnosis and increased mortality in many Asian American subgroups [17,18,19,20,21,22,23,24,25].

Most HBV-related interventional studies have focused primarily on prevention and screening [21,26]. By comparison, very few evidence-based trials have been developed to overcome barriers to HBV monitoring and enhance long-term adherence to disease management in at-risk, underserved Asian American populations. Several studies identified barriers that prevent access to monitoring and treatment resources among Asian Americans living with CHB. These barriers existed not just on the individual level (e.g., financial strains, lack of health insurance, and lack of awareness), but also on cultural and community levels (social stigma and cultural norms around CHB management) and on a systemic level (e.g., lack of translation support in healthcare settings and transportation issues) [27,28,29,30,31]. However, little progress has been made on the development of interventions that help Asian Americans living with CHB overcome barriers to achieve timely and stable access to monitoring and treatment resources.

To address the gaps in literature and practical issues, we designed and implemented a culturally tailored, multicomponent intervention. Through a randomized controlled trial, we sought to determine whether this intervention would be effective in increasing long-term adherence to CHB monitoring guidelines in two Asian American groups—Chinese and Vietnamese—in the greater Philadelphia metropolitan area and New York City area. In this study, we define Asian Americans as persons having origins in any of the original peoples of the Far East, Southeast Asia, or the Indian subcontinent, which is the definition used in the US Census [32]. They could be born in or outside of the US.

## 2. Materials and Methods

From 2019 to 2021, we conducted a two-arm clustered randomized controlled trial to test the efficacy of a culturally tailored, multicomponent intervention in the Greater Philadelphia metropolitan area, which includes Philadelphia and Southern New Jersey. The goal of the intervention was to increase long-term adherence to monitoring and treatment among low-income Asian Americans with CHB. We designed multiple components of the intervention to address several barriers Asian Americans face in accessing CHB monitoring and treatment.

### 2.1. Participant Recruitment

The participants of this study were recruited from two main channels. For the first channel, we recruited from a community-based single-blinded randomized control trial involving Asian Americans with CHB [33]. Specifically, we implemented an interactive patient navigator-led mobile phone text messaging intervention (PNMI) to increase adherence to HBV testing and monitoring among 532 Korean, Chinese, and Vietnamese Americans with CHB (272 in the intervention group and 260 in the control group). From this existing cohort, a total of 240 participants were recruited, including 112 from the intervention group and 128 from the control group. These participants were retained in their respective study arms in the current study. The second recruitment channel was community-based participant recruitment. We conducted outreach to 10 Chinese and Vietnamese American community-based organizations (CBOs) and 5 healthcare providers serving the target populations in the Greater Philadelphia metropolitan area. These CBOs and healthcare providers included those that had already established collaborative relationships with our research team through previous projects, and others that had little or no previous contact with us. From this channel, we recruited 142 participants. We assessed individuals for eligibility on the basis of the following criteria: (1) identify as being of Chinese or Vietnamese descent, (2) at least 18 years old, (3) accessible by cell phone, (4) had been diagnosed with CHB for at least 12 months, (5) had not been compliant with CHB monitoring and treatment for at least 6 months, and (6) not enrolled in other CHB management interventions. These 142 participants were randomly assigned to the two arms. An independent biostatistician not part of this study performed randomization in Microsoft Excel. A block randomization with block size 10 was used to ensure balance within each stratum.

**Figure 1:**
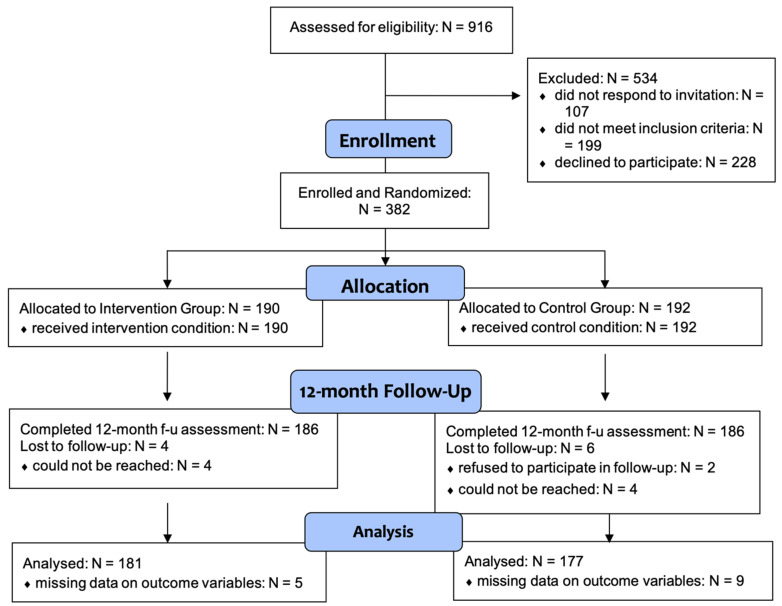
Consolidated Standards of Reporting Trials (CONSORT) Flow Diagram.

Our recruitment efforts through both channels reached a total of 916 Chinese and Vietnamese American men and women aged 18 or older and residing in the Greater Philadelphia area. Among them, 610 met the inclusion criteria, of which 382 consented and completed the baseline assessment. Specifically, 190 were in the intervention group, while 192 were in the control group. Of the 382 participants who completed the baseline assessment, 372 participants completed the 12-month follow-up assessment (intervention group: n = 186; control group: n = 186), while 10 dropped out of the study by the time of follow-up (4 in the intervention group and 6 in the control group). The study retention rate was 97.38% among participants who completed baseline, intervention, and 12-month follow-up (Figure 1). We excluded 14 cases with missing values on key outcome variables and demographic characteristics, which means the analysis sample of this study consisted of 358 individuals (181 in the intervention group and 177 in the control group). All participants received informed consent in the language of their choice, both in writing and via verbal discussion. The study was approved by Western Institutional Review Board, Inc. (WIRB) under the protocol number 20190122.

### 2.2. Intervention Component

**Figure 2:**
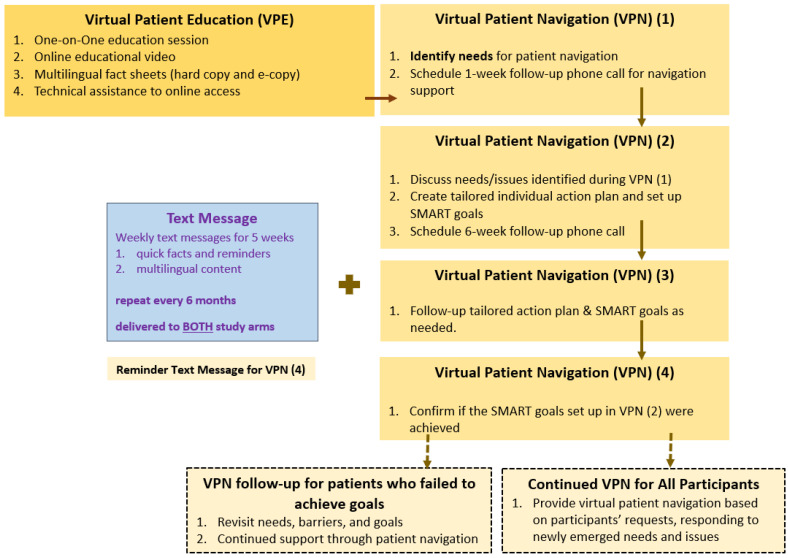
Multicomponent Intervention Flow Chart.

We used the social-ecological perspective [34] and the social cognitive theory [35] as the conceptual framework to guide the design of our culturally tailored, multicomponent intervention. We adopted the community based participatory research (CBPR) approach in the design and implementation of the intervention. Specifically, community leaders, healthcare providers, patient representatives, and other stakeholders were consulted in every step of the process. Notably, patient participants played an active role in the design and delivery of the intervention, including in the virtual patient navigation component of the intervention. Below we detail the three intervention components (Figure 2) and the CBPR strategies used in their design and implementation.

#### 2.2.1. Component 1: Virtual Patient Education (VPE)

We used CBPR-based strategies in the design and implementation of the virtual patient education (VPE) component. Specifically, patients, physicians, and community stakeholders were actively engaged in intervention components development through numerous regular in-person meetings and teleconferences. Stakeholders provided insight in several key aspects of the study design, including in the identification of potential areas of patient needs; in the assessment of language and cultural appropriateness of educational materials; and in the identification of logistical and practical challenges of intervention delivery, feasibility, and accessibility of data collection tools (survey questionnaires).

Based on the suggestions of engaged patients, physicians, and community stakeholders, the contents and literacy levels of the educational materials were upgraded to meet the needs of underserved Asian American HBV patients. Information on CHB monitoring, treatment, and other aspects of management were delivered to the participants in three different formats: presentation slides, fact sheets on CHB management, and educational videos. The presentation slides included 6 modules on CHB management, which covered the importance of managing CHB, monitoring treatment information, managing CHB-related anxiety, improving liver health, accessing financial resources, and protecting families from viral hepatitis. The fact sheets contained information on CHB quick facts, key statistics, and CHB management tips and resources. The educational videos included stories of two Asian American CHB patients sharing personal details about their fight against HBV and liver cancer. The patients talked about their own challenges and successes in managing their CHB.

These educational materials were delivered in two approaches. First, trained bilingual community educators conducted in-person one-on-one sessions with every participant in the intervention group, during which the presentation slides were shown to the participants on an electronic tablet. The slides were provided via email or mail upon participant request. After the one-on-one session was completed, participants received a unique username and password to access all VPE materials in an online educational portal (http://cah-hbvtraining.net/Pages/Login.aspx, accessed on 5 August 2022), where they could conduct further self-directed learning on CHB management through reviewing the presentation slides, fact sheets, and educational videos. This portal allowed participants to refresh their memories, capture more details, and explore topics they may have missed during the one-on-one session. Adjustments made due to the COVID-19 pandemic are detailed later in this article.

#### 2.2.2. Component 2: Virtual Patient Navigation (VPN)

The second component is the virtual patient navigation (VPN). Through providing personalized VPN, participants in the intervention group received assistance in overcoming several barriers to accessing and adhering to CHB monitoring care. VPN was implemented in four steps.

VPN step 1: identifying VPN needs. In the first encounter with a participant, a bilingual virtual patient navigator assisted the participant in identifying individual needs for improving HBV monitoring behaviors. The needs identified included but were not limited to: medication access and adherence support; appointment-making, transportation, and other logistical support; health insurance support; language support; and additional support needs identified by participants. The process of building a good rapport and establishing mutual trust was key in guiding participants in identifying and articulating their needs.

VPN step 2: personalizing a VPN plan and responding to participant needs. Within 1 week after the initial encounter, the patient navigator would initiate a phone call, during which the navigator would address the CHB management needs identified in the first encounter and solicit feedback from participants. Through an interactive process, the navigator and the participant would work together to create a personalized, 6-week action plan using the SMART (Specific, Measurable, Attainable, Realistic, and Time-bound) framework. The patient navigator re-iterated the SMART goals to the participant and scheduled a subsequent follow-up phone call in 6 weeks.

VPN step 3: on-going patient navigation and other assistance via phone calls. The bilingual patient navigators continued to conduct follow-up phone calls to help participants achieve the SMART goals set in the personalized action plan. Adjustments to the plans and supports were constantly made, based on the needs of the participants. Adjustments included responding to the difficulties in accessing CHB care that was exacerbated by the COVID-19 pandemic.

VPN step 4: 6-week follow-up phone call. The navigator initiated the 6-month follow-up phone call to confirm that participants had achieved their SMART goals. If yes, then the active VPN session would be considered successfully completed. If not, the navigator would repeat the VPN regimen to help the participants overcome barriers they were facing in achieving CHB management goals.

#### 2.2.3. Component 3 (Both Study Arms): Mobile Health (mHealth) Text Messages

The first two components were delivered only to the intervention group. The third component, which centered on text message reminders, was provided to both the intervention group and the control group. In a previous randomized clinical trial conducted by this research team, text message reminders were found to be effective in helping Asian Americans with CHB achieve better adherence to treatment [32]. In the present study, a total of five text messages were developed. The contents of the text messages were designed based on feedback from patient representatives, healthcare providers, and community stakeholders in the previous and current studies. The messages contained quick facts on the importance of CHB management, information on resources, reminders on making an appointment for a CHB clinic follow-up, and medication adherence tips. One text message was sent to participants each week. The five-week text messaging routine was repeated every 6 months and would end with this study. Participants in both study arms received the same text messages in the same order and schedule.

#### 2.2.4. Responding to the COVID-19 Pandemic

The COVID-19 pandemic exacerbated existing access barriers and created new challenges among participants. Appointments for clinical follow-ups and laboratory tests were more difficult—at certain points, almost impossible. The study team quickly responded to the circumstances by incorporating information on telemedicine services, resources for chronic disease management during the pandemic, and digital support in our VPE and VPN components.

### 2.3. Measures

The outcomes were CHB monitoring measures, including CHB clinical follow-up in the past 12 months and CHB laboratory monitoring. Chronic HBV infection can lead to more severe liver disease, such as cirrhosis, liver failure, and liver cancer, without showing any significant symptoms in many cases [36]. Therefore, it is important for CHB patients to adhere to physician guidelines to manage CHB. Healthcare providers are recommended to follow the guidelines published by the Centers for Disease Control and Prevention (CDC) on management of persons with CHB [37]. CHB clinical follow-up was measured with one question: “Did you see a doctor to check your hepatitis B infection condition during the past 12 months?” Possible answers were: “No”, “Yes”, and “I do not remember”. With regard to CHB laboratory monitoring, participants were asked to recall if they had undergone blood testing (HBV DNA, liver function, and alpha-fetoprotein testing) in the past 12 months, with the answer options “No”, “Yes”, and “I do not remember”.

Socioeconomic factors, specifically, participants’ age in years, gender, ethnicity, U.S. residency length, marital status, education level, employment status, annual household income, health insurance coverage, and English-speaking proficiency were included as covariates in the regression models on CHB management outcomes.

### 2.4. Statistical Analysis

We followed three steps in our statistical analysis. First, we compared the intervention and control groups with regard to sample characteristics, using cross-tabulations and the chi-square statistic, if the measurement was categorical, or *t*-test, if the measurement was continuous. Second, we compared the two CHB outcome measures at the 12-month follow-up assessment between the intervention and control groups. Third, we fitted the generalized linear mixed-effects models (GLMMs) to assess the intervention effects on the two CHB outcome measures, while controlling for sociodemographic and health-related characteristics. GLMM allowed us to account for sample clustering by recruitment channel and to generate robust standard efforts for the significance tests. We conducted all statistical analysis in R [38]. A *p* value that is smaller than 0.05 was considered statistically significant.

## 3. Results

The study sample consisted of 358 Asian American adults with CHB within the Philadelphia and NYC metro areas, with a mean age of 53. About 76% of participants were Chinese, with the remaining 24% being Vietnamese. The overall sample had relatively low socioeconomic standing, with half of the sample reporting an annual household income lower than USD 20,000. About 99% of participants were born outside of the U.S., with more than two-thirds of the total sample reporting their English proficiency as “not at all/not well”. The intervention and control group did not significantly vary on any of the socio-demographic characteristics presented in Table 1.

To examine the intervention effects, a generalized linear mixed-effect regression was applied to the two outcome variables, CHB clinic follow-up (Table 2) and CHB laboratory monitoring (Table 3), at 12-month follow-up assessment. Odds ratio (OR) and 95% confidence intervals (CIs) were presented. For each outcome, two models were fitted: Model 1, with only the study arm regressed on the outcome, and Model 2, with other covariates controlled for in the model. The intervention group had a substantially higher CHB clinic follow-up rate (86.2%) and CHB laboratory monitoring (79.0%) at the 12-month follow-up assessment than did the control group (54.2% and 45.2%, respectively). The generalized linear mixed-effect regression results showed that the intervention group was significantly more likely than the control group to have visited their doctor for CHB (OR = 7.35; 95% CI = 4.06–13.33; *p* < 0.001) and to have undergone blood testing (OR = 6.60; 95% CI = 3.77–11.56; *p* < 0.001).

## 4. Discussion

The Viral Hepatitis National Strategic Plan for the United States has called for improved implementation of recommended monitoring and care for individuals with CHB to prevent morbidity and mortality from HCC, end-stage liver disease, and other hepatitis-related sequelae [39]. Treatment effectiveness diminishes if patients with CHB do not adhere to long-term monitoring and antiviral treatment guidelines [3,4]. Inadequate monitoring and care contribute to poor outcomes and increased CHB-associated healthcare costs, which are estimated to be as high as USD 1.5 billion per year [7,40]. Therefore, helping patients overcome the multilevel barriers they face in accessing monitoring and treatment care over the long term is crucial in reducing liver disease and liver cancer burden.

As demonstrated in this study, a community-based culturally appropriate intervention had significant effects on whether participants underwent CHB clinical follow-up for their CHB and whether they completed the appropriate CHB laboratory monitoring in a 12-month period. Our results showed a large intervention effect, similar to a previous education intervention in a multi-ethnic safety-net population in California [41]. In this study, researchers found that the HBV educational intervention was associated with significantly higher odds of appropriate CHB clinic follow-up (OR = 7.02; 95% CI = 3.64–13.56; *p* < 0.001) and appropriate laboratory monitoring (OR = 4.94; 95% CI = 2.64–9.25; *p* < 0.001). Our intervention had slightly larger odds ratios on CHB clinic follow-up (OR = 7.37) and laboratory monitoring (OR = 6.60).

The multicomponent, language congruent intervention of the present study was implemented with a CBPR approach. Community leaders, stakeholders, health providers, and patient representatives were engaged in every stage of the project, including intervention design, delivery, and evaluation. They provided critical insight on the cultural appropriateness of the study design and implementation process and played key roles in ensuring the success of participant recruitment, participant retention, and intervention efficacy on long-term CHB monitoring outcomes. This study adds to the growing evidence of the importance of the CBPR approach in health promotion among racial/ethnic minority groups [21,42,43].

While the self-reported outcomes were potentially affected by the social desirability effect, our previous research of similar topics used medical records as validation and found little discrepancy between clinical follow-up and laboratory monitoring behaviors and self-reported behaviors among low-income Asian Americans with CHB [33].

The success in increasing CHB monitoring adherence among Asian American patients may be attributed to the combined effects of increasing community awareness, improved management of emotional well-being, and raised logistical support via VPN [21,44,45]. Future research should better assess the effects of each individual component and better delineate the specific psychosocial mechanisms of behavioral changes relevant to improving long-term CHB monitoring outcomes.

In conclusion, the present study contributes to the field of population-based health disparities research by providing strong evidence that a culturally appropriate, multicomponent intervention addressing individual- and systemic-level barriers in CHB healthcare access can significantly benefit health behavior among Asian Americans living with CHB [21,44,45]. Such interventions, with tailoring for cultural relevance, could prove applicable to other racial/ethnic groups that are disproportionately affected by CHB.

## Figures and Tables

**Table 1:** Sociodemographic and health-related characteristics of the sample, in total and by study arm.

	Intervention (N = 181)	Control (N = 177)	Total (N = 358)	*p* Value
	n (%)	n (%)	n (%)	
Participant Source				0.14
existing cohort	106 (58.6%)	117 (66.1%)	223 (62.3%)	
new participants	75 (41.4%)	60 (33.9%)	135 (37.7%)	
Age in years				0.99
mean (SD)	53.18 (12.63)	53.20 (13.93)	53.19 (13.27)	
Ethnicity				0.70
Chinese	138 (76.2%)	138 (78.0%)	276 (77.1%)	
Vietnamese	43 (23.8%)	39 (22.0%)	82 (22.9%)	
Gender				0.66
male	87 (48.1%)	81 (45.8%)	168 (46.9%)	
female	94 (51.9%)	96 (54.2%)	190 (53.1%)	
Marital Status				0.37
currently married	145 (80.1%)	145 (83.8%)	290 (81.9%)	
not married	36 (19.9%)	28 (16.2%)	64 (18.1%)	
Education				0.67
≤hs	123 (68.0%)	124 (70.1%)	247 (69.0%)	
≥college	58 (32.0%)	53 (29.9%)	111 (31.0%)	
Employment				0.24
employed	115 (64.2%)	109 (61.9%)	224 (63.1%)	
unemployed	9 (5.0%)	17 (9.7%)	26 (7.3%)	
not in labor force	55 (30.7%)	50 (28.4%)	105 (29.6%)	
Annual Household Income				0.17
<USD 20 k	85 (47.0%)	96 (54.2%)	181 (50.6%)	
≥USD 20 k	96 (53.0%)	81 (45.8%)	177 (49.4%)	
Nativity Status				0.17
foreign-born	180 (99.4%)	173 (97.7%)	353 (98.6%)	
US-born	1 (0.6%)	4 (2.3%)	5 (1.4%)	
Length of U.S. Residency				0.35
<10 yrs	20 (11.5%)	25 (14.9%)	45 (13.2%)	
≥10 yrs	154 (88.5%)	143 (85.1%)	297 (86.8%)	
Having Health Insurance				0.04
no	20 (11.0%)	33 (18.8%)	53 (14.8%)	
yes	161 (89.0%)	143 (81.2%)	304 (85.2%)	
Having A Regular Physician				0.16
no	20 (11.6%)	27 (17.0%)	47 (14.2%)	
yes	152 (88.4%)	132 (83.0%)	284 (85.8%)	
English Proficiency				0.07
not at all/not well	116 (64.1%)	129 (72.9%)	245 (68.4%)	
well/very well	65 (35.9%)	48 (27.1%)	113 (31.6%)	

**Table 2:** Odds Ratio of CHB Clinical Follow-up at 12 Months of the Intervention Group Against the Control Group, Results from Descriptive Analysis and Generalized Linear Mixed-Effect Models.

Variable	Total	Intervention	Control
No. participants	358	181	177
CHB clinic follow-up, %		86.2%	54.2%
Model 1, OR (95% CI)		5.21 (3.11–8.74)	1 (Ref)
Model 2, OR (95% CI)		7.35 (4.06–13.33)	1 (Ref)

Note. OR = odds ratio; CI = confidence interval; Model 1 included only the study arm regressed on the outcome; Model 2 controls for recruitment source (existing cohort vs. new recruits), age, gender, education, marital status, ethnicity, employment status, annual household income, health insurance access, access to regular physician, U.S.-Born, English Proficiency.

**Table 3:** Odds Ratio of CHB Laboratory Monitoring at 12-Month Follow-Up of the Intervention Group Against the Control Group, Results from Descriptive Analysis and Generalized Linear Mixed-Effect Models.

Variable	Total	Intervention	Control
No. participants	358	181	177
CHB laboratory monitoring, %		79.0%	45.2%
Model 1, OR (95% CI)		4.53 (0.59–7.22)	1 (Ref)
Model 2, OR (95% CI)		6.60 (3.77–11.56)	1 (Ref)

Note. OR = odds ratio; CI = confidence interval; Model 1 included only the study arm regressed on the outcome; Model 2 controls for recruitment source (existing cohort vs. new recruits), age, gender, education, marital status, ethnicity, employment status, annual household income, health insurance access, access to regular physician, U.S.-Born, English Proficiency.

## Data Availability

The data presented in this study are available on request from the corresponding author.
